# Cellular mechanisms linking to outdoor and indoor air pollution damage during pregnancy

**DOI:** 10.3389/fendo.2023.1084986

**Published:** 2023-02-15

**Authors:** Delia I. Chiarello, Javier Ustáriz, Reinaldo Marín, Ivo Carrasco-Wong, Marcelo Farías, Ady Giordano, Felipe S. Gallardo, Sebastián E. Illanes, Jaime Gutiérrez

**Affiliations:** ^1^ Cellular Signaling and Differentiation Laboratory (CSDL), School of Medical Technology, Faculty of Medicine and Science, Universidad San Sebastián, Santiago, Chile; ^2^ Department of Chemical and Bioprocess Engineering, Pontificia Universidad Católica de Chile, Santiago, Chile; ^3^ Center for Biophysics and Biochemistry (CBB), Venezuelan Institute for Scientific Research (IVIC), Caracas, Venezuela; ^4^ Faculty of Medicine, Pontificia Universidad Católica de Chile, Santiago, Chile; ^5^ Inorganic Chemistry Department, Faculty of Chemistry and of Pharmacy, Pontificia Universidad Católica de Chile, Santiago, Chile; ^6^ Reproductive Biology Program, Center for Biomedical Research and Innovation (CiiB), Universidad de los Andes, Santiago, Chile; ^7^ IMPACT, Center of Interventional Medicine for Precision and Advanced Cellular Therapy, Santiago, Chile; ^8^ Department of Obstetrics and Gynecology, Faculty of Medicine, Universidad de los Andes, Santiago, Chile

**Keywords:** exposome, pregnancy, indoor - outdoor pollution, mitigation strategies, cell damage, PM_2.5_

## Abstract

Pregnancies are a critical window period for environmental influences over the mother and the offspring. There is a growing body of evidence associating indoor and outdoor air pollution exposure to adverse pregnancy outcomes such as preterm birth and hypertensive disorders of pregnancy. Particulate matter (PM) could trigger oxi-inflammation and could also reach the placenta leading to placental damage with fetal consequences. The combination of strategies such as risk assessment, advise about risks of environmental exposures to pregnant women, together with nutritional strategies and digital solutions to monitor air quality can be effective in mitigating the effects of air pollution during pregnancy.

## 1 Introduction

Urban daily exposures to chemical mixtures originated from air pollution have a profound effect on health, especially during vulnerable periods of development such as intrauterine life (1, 2). Pregnancy exposome has been emerging as a focal point in developmental origins of diseases, because of its ability to influence the epigenome and thus to affect gene activity and expression, modifying the likelihood of maternal comorbidities and perinatal outcomes risk (2–4). The exposome, defined as the totality of environmental exposure of an individual over the lifespan, is proposed to complement the genome information since the exposome is highly variable and dynamic (5). The exposome includes the general external environment factors, for example, environmental pollution, climate, and sociodemographic factors (6). According to the World Health Organization (WHO), air pollution (indoor and outdoor) “represents the single largest environmental risk to health globally” (7). Moreover, exposure to air pollutants is emerging as another key factor to determine the susceptibility of an adverse pregnancy outcome (8).

Fetal development is a critical window for every single mammal on earth, including human beings (9). During this period, the embryos depend on the health, nutrition, activities, and emotional status of the mother, which can modify the fetal exposome (2). According to Barker’s theory, embryonic development determines the physiological and metabolic responses that the individual will have into adulthood, in a process known as fetal programming. Thus, any stimulus or insult during embryonic development will result in developmental adaptations that produce permanent structural, physiological, and metabolic changes that predispose to cardiovascular, metabolic, and endocrine disease in adult life (10). Epidemiological data associates air pollution exposure during pregnancy with adverse outcomes such as preterm birth (<37 weeks of gestation) (11, 12), low birth weight (< 2500 g at birth) (13), miscarriage (14), preeclampsia and hypertensive disorders of pregnancy (15). Although the mechanisms responsible for the adverse pregnancy outcomes related to air pollution are not elucidated, recent experimental evidence indicates that the placenta is a direct target tissue for air pollution (16, 17). It is suggested that oxidative stress, endocrine disruption, inflammatory response, and DNA damage are the main contributors (18, 19). The immature metabolism and cellular proliferation period that characterizes the growing fetus is highly vulnerable to pollutants exposure (20).

The aim of this review is to provide an updated overview of the evidence linking exposure to outdoor or indoor air pollution during pregnancy with effects at the cellular level and some ways to mitigate these effects will also be discussed.

## 2 Air pollution

Air pollution is defined as environmental contamination by toxic chemical compounds, gases, and particles that could modify the natural characteristics of the atmosphere with potential adverse health effects (21). Air pollution encompasses a mixture of different pollutants including particulate matter (PM), among many sources of PM, the combustion emissions from fossil fuel engines and degradation of vehicle parts and road surfaces abrasions represent substantial contributions (22). PM are defined according to their aerodynamic diameter as ultrafine particles PM_0.1_ (median aerodynamic diameter <0.1 μm), fine particles PM_2.5_ (median aerodynamic diameter <2.5 μm), coarse particles PM_10_ (median aerodynamic diameter <10 μm) and gaseous components like ozone (O_3_), carbon monoxide (CO), sulfur dioxide (SO_2_), nitrogen dioxide (NO_2_), carbon dioxide (CO_2_), black carbon (BC), diesel exhaust fumes, a wide variety of toxic chemicals such as polycyclic aromatic hydrocarbons (PAHs) and volatile and semi-volatile organic compounds (VOCs) (23, 24). Air pollution is a dynamic phenomenon where both outdoor and indoor pollutants interact and affect air quality (25).

The particulate matter often derives from a different source and has different chemical compositions. PM_10_ commonly includes pollen debris, dust from landfills or construction sites, industrial sources, wildfires, brush or waste burning, wind-blown dust from open lands, and microbial fragments (26). PM_0.1_ and PM_2.5_, often linked to biomass and traffic combustion (27, 28), are relevant in rural and urban environments, since they can be inhaled deeply into the lungs and enter the bloodstream, increasing the potential risk for cardiovascular diseases (29), lung cancer (30) and adverse pregnancy outcome (31). PM_2.5_ is composed of a mixture of natural crustal materials (carbonates, silicates), metals (copper, arsenic, and vanadium), inorganic molecules (sulfate, nitrate, sodium, potassium, and ammonium), black carbon and organic compounds (such as polycyclic aromatic hydrocarbons) (32). Polycyclic aromatic hydrocarbons (PAHs) are a large family of organic compounds, formed as products of incomplete combustion from natural (fly ash and soot from wood burning) and anthropogenic sources (motor vehicle exhaust, tobacco smoking, industrial processes, demolition waste), and are often present in the air, soil, and water (33). PAHs are comprised of two or more fused benzene rings in different arrangements (linear, clustered, and angular) highly lipophilic with relatively low solubility in water, that are stable and resistant to hydrolysis (34). Ambient air is one of the major sources of PAHs exposure (35). In the atmosphere, most PAHs present on PM_2.5_ have low volatility (particle-phase), which is characteristic of PAHs containing five or six aromatic rings such as benzo[b]fluoranthene, benzo[g,h,i]perylene, and benzo[a]pyrene (B[a]P). These compounds are linked to mutagenic, teratogenic, and carcinogenic properties (34).

PAHs have short half-lives in the blood (36). Inside the cells, the first phase of biotransformation starts with the recognition of the PAHs by the aryl hydrocarbon receptor (AhR), the complex PAH-AhR is translocated into the nucleus and induces cytochrome P450 *(Cyp)1a1* gene expression, that encodes for xenobiotic-metabolizing enzyme CYP1A. The specific metabolites (several phenols) can bind DNA and form PAH–DNA adducts (37). In the second phase of biotransformation, the resulting phenolic compounds from the metabolization reactions are conjugated to glutathione, glucuronides, and sulfate esters to enhance the aqueous solubility to finally be excreted in urine (38). B[a]P (five-ring) is a member of the PAH family that can accumulate in the placenta (39, 40) inhibiting trophoblast cells differentiation and proliferation (41), disrupting the endocrine placental function (42), disturbing the redox balance (39) and forming DNA adducts (43).

## 3 Air pollution exposure during pregnancy

### 3.1 Pregnancy outcomes and outdoor air pollution

Outdoor air pollution exposure during pregnancy has been linked to fetal development problems, preterm birth, and pregnancy complications, including pregnancy-induced hypertensive disorders (44–49). In general, outdoor pollutants refer to exhaust emissions from vehicle emissions, however, in recent years these emissions have been significantly reduced, especially in developed countries (50). While non-exhaust PM emissions have gained interest in the developed countries (51), these emissions are generated by clutch and engine wear, abrasion of wheel bearings, corrosion of other vehicle components, street furniture, crash barriers or resuspension of road dust has been rising (52). Only a few studies distinguish between exhaust and non-exahust airbone particles. Regarding this, a retrospective population-based cohort study performed in 540 365 singleton births used two pollutant models including source specific PM_2.5_ and found that the magnitude of the association between low birth weight (LBW) and the exhaust PM_2.5_ component was consistently stronger than with non-exhaust PM_2.5_ (53). A cohort study with 34,705 singleton births delivered at Pittsburgh, between 1999 and 2002, reports the association between preeclampsia, gestational hypertension, and preterm delivery with increased exposure to an ambient source of PAHs (PM_2.5_) during the first trimester of pregnancy (54). Non-exhaust emissions contribute primarily to PM_10_ and to a lesser extent PM_2.5_ however the effects have been less explored compared to those of exhaust PM (55). PM_10_ has been associated with fetal overgrowth (56), with preeclampsia, particularly during humid periods (autumn/winter seasons) (57); conversely PM_10_ has also been associated with small for gestational age (a birth weight of less than 10th percentile for gestational age) in twins born between 32 and 36 weeks, but not associated in term twins (58). Suggesting that PM10 increases the risk of abnormal fetal growth (59). Additionally, it has been reported that exposure to PM_10_ during the first trimester can alter the fetal heart response rate without evidence of acidemia or fetal asphyxia (60). Regarding other compounds, several studies have concluded that outdoor SO_2_ exposure during the third trimester was associated with early-term births (birth between 37 and 38 weeks) (61). While the associations for NO_2_, NO, CO and O_3_ were inconclusive in some cases (62), in other cases NO_2_ was linked to preterm birth, even among pregnant women living in an area with relatively low average air pollution concentrations (63) and consistently associated with term low birth weight (64). On the other hand, a systematic review of 84 studies found that most types of particulate matter (PM) were associated with low birth weight, but these associations had many inconsistencies in terms of PM sources and the characteristics of the built environment, proximity to traffic, and green spaces near the residence of the pregnant women (65). A recent study that analyzed the records of almost 600,000 pregnant patients demonstrated a positive association between preterm birth and PM_10_, PM_2.5_, SO_2_, NO_2_ and CO, where NO_2_ was the largest pollutant contributor while the third trimester was identified as the most sensitive exposure window (66). Meanwhile, a recent report showed a positive association between three-month mean residential NO_2_ concentrations and maternal hair cortisol as a biomarker for longer-term biological stress during pregnancy (67). Another meta-analysis that included more than 60 studies found that exposure to PM_2.5_, PM_10_, and O_3_ during pregnancy correlates with the risk of preterm birth at 32-35 weeks, 28-31 weeks, and before 28 weeks (68). Exposure to PM_2.5_ pollution during pregnancy is significantly associated with preeclampsia and hypertensive disorders of pregnancy (69). Preeclampsia is a pregnancy pathology associated with placental dysfunction and defined by a new onset of hypertension with or without proteinuria after 20 weeks of gestation (70). A recent meta-analysis performed with data up to March 2020, summarized 9 cohort studies and concluded that maternal exposure to PM_2.5_ during the third trimester of pregnancy elevates the risk of preeclampsia (71). Some epidemiological studies, use the distributed lag linear model, which is a statistical analysis model that distributes the effect of a single exposure event over a specific time period, in order to estimate the lag effect between exposure to ambient air pollutants and a health outcome (72). During pregnancy, several studies have analyzed the lag effect of different air pollutants exposure and its association with the risk of adverse pregnancy outcomes for example, a study reported a significant association between preconceptional air pollution exposure (PM_2.5_, PM_10_, and O_3_) during the cold season and the termination of pregnancy (73). A recent study applied distributed lag nonlinear model to investigate the association between early pregnancy to midpregnancy exposures to PM_2.5_, PM_10_, and NO_2_ and lower birth weight (74). Another study found that the acute and lag effects of high levels PM_2.5_, PM_10_, NO_2_, and SO_2_ exposure of the calculated fertilization time was associated with spontaneous abortion preterm birth (75). The identification of critical windows of susceptibility in which exposure to air pollutants may alter pregnancy outcome is key to improve environmental health interventions and prevent vulnerable populations, currently remains inconsistent across studies, further research is needed to investigate the most likely window of exposure, as well as to estimate the lag and acute effect of exposure to air pollutants during pregnancy (76).

### 3.2 Maternal physiological adaptation during pregnancy and particulate matter exposure

Normal pregnancy implies profound cardiovascular changes necessary to meet the increased demands of the growing fetoplacental unit (77). These changes occur very early in pregnancy, then, the exposure to PM_2.5_ in this period could have a detrimental effect on cardiovascular adaptations during pregnancy leading to the development of hypertensive disorders (78). It has been shown that the smaller particles are more harmful than the larger particles inducing adverse health effects, as the deposition rate is strongly influenced by particle size (79). Fine particles (PM2.5) are stable in the atmosphere with a residence time of 7 to 30 days, being wet deposition by precipitation the predominant removal mechanism (80), in addition dry deposition by gravitational settling has been reported (81). Recent work showed an association between the PM_2.5_ exposure during the first trimester and the development of hypertensive disorders of pregnancy (82) and maternal thyroid dysfunction (75). Since pregnancy is associated with maternal respiratory adaptations, mainly related to significantly increasing tidal volume, pregnant women could inhale more polluted air (83). The acute or chronic exposure to PM_2.5_ causes activation of inflammatory responses (**Table 1**) together with structural damage to the alveoli, which facilitates the passage of PM_2.5_ into the systemic circulation (84, 104).

**Table 1:** Cellular effects associated to air pollution exposure during pregnancy.

Reference	Study design	Exhaust/non-exhaust	Pollutants associate to observed effects	Exposure concentration/time associate to observed effects	Observed effects	Statistics of associations
Smith et al. (53)	Retrospective population-based cohort study. 540 365 singleton term live births	exhaust non-exhaust	PM_2.5traffic exhaust_ from London, UK PM_2.5traffic non-exhaust_ from London, UK	>13.8 μg/m^3^	Residential exposure to PM_2.5_ traffic exhaust during pregnancy is directly attributable to 3% of term LBW cases in London By analogy, the authors hypothesize that the mechanisms involved may include, oxidative stress, changes in oxygen or nutrition transfer, placental mitochondrial damage, or endocrine disruption	Odds ratio (95% CI) PM_2.5traffic exhaust_ 1.04 (1.01 to 1.07)
Zhou et al. (19)	Cohort of 1060 mother-child pairs recruited between April 2016 and December 2018 Sample: maternal blood	mixture	PM_2.5_ from Shanghai, China	Mean 37.74 μg/m^3^	PM_2.5_ inorganic constituents, such as Al, Si, K, Mn, and Zn, appear to be responsible for increase in maternal TSH and decreased in maternal serum free thyroxine (fT4) that lead changes in fetal growth measures	% change (95% CI) Maternal TSH: increases of 12.75% (1.01%, 24.61%) Maternal fT4: decreases of 5.82% (8.61%, -2.96%)
Liu et al. (84)	Cell line: Epithelial alveolar cells (A549)	mixture	PM from urban dust (SRM1649b) purchased from NIST (MD, USA)	100 μg/mL/24 h	Increases the expression of ROS, ICAM-1 and the production of interleukin-6 (IL-6)	n.a
Bové et al. (17)	20 mothers Sample: Placenta	exhaust	BC from Belgium	annual means ranging from 0.63 to 2.42 µg per m^3^/entire pregnancy	The presence of particles from air pollution within the placenta of all participating mothers was evidenced. The number of placental particles per mm^3^ was positively associated with the mothers’ residential exposure to BC during pregnancy	Increases (95% CI) Increased placental BC load associated with increased residential BC exposure:+0.45 × 10^4^ per mm^3^ (0.11 × 10^4^ to 0.80 × 10^4^)
Li et al. (85)	Cell line: HAEC	exhaust	Diesel exhausted particles, collected from a 1998 Kenworth truck	50 μg/ml/4 h	Down-regulation of Zonular Occludin-1 (ZO-1) leading an increase in permeability	n.a
Le et al. (86)	Primary cell culture: HUVEC	mixture	PM_2.5_, China	10 μg/mL/12 h	Increases mRNA and protein levels of TLR2, TLR4 Increase mRNA expression of IL-1 *β*, and IL-6	n.a
Su et al. (87)	Primary cell culture: HUVEC	mixture	PM_2.5_ from Taiyuan, China	10 μg/cm^2/^6 h	Induced significant structural and functional damage in mitochondria and lysosomes PM_2.5_ internalization is mediated by clathrin and caveolin	n.a
Ma et al. (88)	Cell line: MHC	mixture	PM_2.5_ from Langfan, China	500 μg/mL/48 h	PM_2.5_ induces Endothelial–Mesenchymal Transition (EndMT) by activating the TGF-β1/Smad3/p-Smad3 pathway.	n.a
Xu et al. (117)	Primary cell culture: HUVEC	mixture	PM_2.5_ from Wuhan, China	12.5 μg/mL/24 h	The activation of ERKs, p38 kinase and JNKs mediates the induction of AT1R	n.a
Xu et al. (89)	Primary cell culture: HUVEC	mixture	PM_2.5_ from Wuhan, China	12.5 μg/mL/24 h	Induction of endoplasmic reticulum stress leading to HIF1α transactivation, which in turn mediates endothelial dysfunction by upregulation of components of the ACE/ANGII/AT1R axis in the endothelial cell	n.a
Wauters et al. (90)	12 healthy male volunteers	exhaust	PM_2.5_ from diesel exhaust from PSA DW10 engine (common in Europe)	300 μg/m^3^/120 minutes at rest	The acetylcholine/sodium nitroprusside vasodilation ratio decreased after polluted air decreased significantly and was inversely correlated to the total amount of PM_2.5_ inhaled. Impairments in microvascular function measured by NO bioavailability decrease and ROS increase.	Spearman correlation coefficient (r=−0.55, P<0.01)
Calderón-Garcidueñas et al. (91)	Healthy children, 6–13 years of age	mixture	PM_2.5_ from Mexico City	4000 μg/m^3^/h/7-day cumulative outdoor dose	The increase in circulating ET-1 concentrations showed a positive association with the number of daily hours outdoors. An increase in mean pulmonary arterial pressure (PAPM) was observed.	Pearson’s correlation (r = 0.31, p = 0.012)
Finch et al. (118)	Young, healthy nonsmokers	mixture	PM_2.5_ from Utah, USA	50 μg/m^3^/24 h	Negative association between acute exposure to PM_2.5_ and circulating levels of ET-1	Beta (95% CI) ambient PM_2.5_ and blood ET-1: β −0.773 (−1.18, −0.365)
Grevendonk et al. (92)	293 mother-newborn pairs	mixture	PM_2.5_ and PM10, from Belgium	PM_2.5_, 16.6 μg/m^3/^entire pregnancy PM_10_, 21.4 μg/m^3^/entire pregnancy	PM_10_ and PM_2.5_ exposure during the entire pregnancy were positively correlated with mitochondrial 8-OHdG levels in maternal blood	% change (95% CI) PM_2.5_ exposure: increase of mitochondrial 8-OHdG levels in maternal blood 13.9% (0.4 to 29.4%) PM_10_ exposure: increase of mitochondrial 8-OHdG levels in maternal blood 18.3% (5.6 to 33.4%)
Saenen et al. (93)	502 mother-newborn pairs	mixture	PM_2.5_ and BC from Belgium	PM_2.5_, 15.8 μg/m^3/^first trimester of pregnancy PM_2.5_, 15.3 μg/m^3/^second trimester of pregnancy BC, 0,90 μg/m^3/^first trimester of pregnancy	Placental nitrosative stress marker, 3-nitrotyrosine (3-NTp) were positively associated with PM_2.5_ and BC exposure levels during gestation	% change (95% CI) Placental 3-NTp increases by each gestational time window of exposure PM_2.5_ first trimestre exposure: 29.0%, (4.9, 58.6); second trimestre exposure: 39.3%, (12.3, 72.7) BC first trimester exposure: 23.6%, (4.4, 46.4)
Dong et al. (40)	64 pregnant women	exhaust	_HMW_PAHs compounds, mainly from the incomplete combustion or pyrolysis of biomass from Kunming, China	n.a.	The low accumulation of PAHs inside the placenta was related to pregnancy complications and increased levels of PAHs in maternal and umbilical cord blood.	n.a.
Familari et al. (94)	Cell line: HTR-8/SVneo cells	mixture	PM_2.5_ from Malmö, Swedenand PM_10_ from Prague, Czech Republic	500-5000 ng/mL/48h	Decreased hCGβ secretion and increased IL-6 secretion	n.a.
Nääv et al. (95)	Cell line: HTR-8/SVneo cells	mixture	PM_2.5_ from Malmö, Sweden	500 ng/mL/48h	Cytotoxicity, increase in progesterone and IL-6 secretion	n.a.
Qin et al. (96)	Cell line: HTR-8/SVneo cells	mixture	PM_2.5_ from Tianjin City, China	120 μg/mL/24 and 48 h	Inhibition of migration and invasion, DNA damage and cell cycle G2/M arrest Higher ROS generation and increasing TIMP1 and TIMP2 expression	n.a.
Agarwal et al. (39)	84 pregnant women Sample: Placental tissue	exhaust	PAHs from Agra, India	_LMW_PAHs 2.047 μg/L/n.a _HMW_PAHs 3.016 μg/L/n.a total PAHs 5.064 μg/L/n.a	Negative correlation was observed between low, high, total PAHs and GSH levels, both in placental tissue. The level of MDA was significantly high in placental tissue and was associated with total PAHs levels. The observed increase in MDA and decrease in GSH suggests an imbalance in oxidant homeostasis	Pearson’s correlation GSH and _LMW_PAHs (r = -0.306, p < 0.01), _HMW_PAHs (r = -0.441, p < 0.001), and TPAHs (r = -0.388, p < 0.001) MDA and total PAHs (r = 0.27, p = 0.0128).
Herbstman et al. (97)	164 pregnant women maternal Sample: umbilical cord blood leukocytes	exhaust	PAHs from New York City, USA	PAHs, including pyrene, 5.314 ng/m^3^/ third trimester	Maternal exposure to PAHs decreased cord blood global methylation however, B[a]P–DNA adduct formation was associated with higher global DNA methylation in umbilical cord white blood cells	β-values (95% CI) Global methylation in umbilical cord blood and prenatal PAHs exposure: β = –0.11; (–0.21, 0.00) Odds ratio (95% CI) BaP-DNA adducts in umbilical cord blood and increased levels of genomic methylation 2,35; (1,35, 4,09)
Al-Saleh et al. (38)	1578 women Sample: maternal urine and placental tissue	exhaust	PAHs from Al-Kharj, Saudi Arabia	PAHs n.a.	High levels of BaP in the placenta were associated with decreased placental thickness and decreased cord length. A positive relationship was found between the levels of 8-OHdG and 1-HP in maternal urine.	β-values (P) Placental thickness −0.071 (0.018) Cord length −0.074 (0.013) β Weight (P) 8-OHdG and 1-HP 0.303 (<0.001)
van Drooge et al. (98)	Cell line: JEG-3 cells	mixture	Rural and urban PM_1_ from Barcelona, Spain	PM_1_ 11 and 12 m^3^ eqAir/mL/24 h	PAHs from PM1 emitted by biomass burning induce cytotoxicity and inhibition of aromatase activity in JEG-3 cells	n.a
Karttunen et al. (43)	12 uncomplicate pregnancies Tissue: Placenta	exhaust	^3^ H-BP (Amersham Biosciences)	0.1 – 1 μM BP/15 min – 6 h	Transfer of BP from the placental maternal side to the fetal circulation was confirmed by placental perfusion experiments BPDE–DNA adducts were found in placental tissue after the perfusion with 1 μM BP	n.a
Wang et al. (99)	Cell line: Swan71 cells	exhaust	Benzo(a)pyren-7,8-dihydrodiol-9,10-epoxide (BPDE)	0.25 – 4 μM BPDE/24 h	BPDE reduces hCG secretion and also prevents trophoblast cell invasion in a dose-dependent manner BPDE induces apoptosis in a dose-dependent manner and induced mitochondrial damage BPDE increase in ROS, MDA, and inflammation, and decrease in SOD	n.a
Wang et al. (100)	Cell line: JEG-3 cells	mixture	PM_2.5_ from Shanxi, China	1 - 10 μg/mL/48 – 96 h	JEG-3 cells exposure to PM_2.5_ increased hCG levels at both 24 h and 48 h Cell proliferation decreased at 24 h	n.a
Wakx et al. (101)	3 healthy mothers with uncomplicated pregnancies Primary cell culture: Ex vivo trophoblasts cells Cell line: JEG-3	exhaust	B[a]P (Sigma, Saint-Quentin Fallavier, France)	0.1 – 10 μM/72 h	In ex vivo placental cells, incubation with 10 μM B[a]P for 48 h did not cause loss of cell viability or DNA fragmentation. JEG-3 cells exposed to 10 μM B[a]P for 72 h leads to cell cycle arrest (G2/M phase) and a significant decrease in cell proliferation and DNA damage.	
Pidoux et al. (102)	Tissue: Placenta	n.a	[14C]-formaldehyde (50 mCi/mmol, Perkin-Elmer)	100 μM	Accumulation of formaldehyde in the placenta and the fetal compartments and hormonal dysfunction	n.a
Shen et al. (103)	HUVECs	n.a	Cooking Oil Fumes-derived PM_2.5_	25, 50, 100, 150, and 200 μg/mL	Reduce cells viability, overproduction of ROS, activation of NLRP3 and IL-1β inflammasome, and inhibition of VEGF expression which directly affects angiogenesis	n.a

UK, United Kingdom; LBW, Low birth weight; TSH, thyroid-stimulating hormone; fT4, free thyroxine 4; ROS, Reactive oxygen species; ICAM, Endothelial adhesion molecule; BC, Black carbon; HAEC, Human aortic endothelial cells; HUVEC, Human umbilical vein endothelial cells; TLR2, Toll Like Receptor 2; TLR4, Toll Like Receptor 4; IL-1 β,  Interleukin 1β; IL-6, Interleukin 6; MHC, Mouse pulmonary microvascular endothelial cells; TGF-β1, Transforming growth factor-β 1; Smad3, Mothers against decapentaplegic homolog 3; p-Smad3, Phospho-Smad3; ERK, Extracellular signal regulated protein kinase; p38, Family is a highly evolutionarily conserved group of mitogen-activated protein kinases; JNK, c-Jun N-terminal kinase; AT1R, Angiotensin II type 1 receptor;  HIF1α, hypoxia inducible factor 1 subunit alpha; ACE, Angiotensin-Converting enzyme; ANGII, Angiotensin II; PSA DW10, diesel engine manufactured by Peugeot S.A; NO, Nitric oxide; ET-1, Endothelin 1; 8-OHdG, 8-Hydroxy-2'-deoxyguanosine; 1-HP, 1-hydroxypyrene; HMWPAHs, High-molecular-weight polycyclic aromatic hydrocarbons; HTR-8/SVneo,  Immortalized first trimester human trophoblast cells; hCGβ, Human chorionic gonadotropin β; TIMP1, Tissue inhibitor of metalloproteinase 1; TIMP2, Tissue inhibitor of metalloproteinase 2; G2/M arrest, Cell cycle arrest at the G2/M phase occurs when DNA is damaged; PAHs, Polycyclic Aromatic Hydrocarbons; GSH: Glutathione; LMWPAHs, Low-molecular-weight polycyclic aromatic hydrocarbons; MDA, malondialdehyde; B[a]P–DNA or BaP-DNA, DNA binding by Benzo[a]pyrene; B[a]P or BaP, Benzo[a]pyrene; JEG-3 cells, Human choriocarcinoma cell line; 3H-BP, 3H-benzo(a)pyrene (BP); BPDE-DNA, DNA binding by Benzo(a)pyrene diolepoxide; Swan71 cells, Immortalized human trophoblast cells;  hCG, Human chorionic gonadotropin; SOD, Superoxide dismutase; NLRP3, NOD-, LRR- and pyrin domain-containing protein 3; IL-1β, interleukin-1beta; VEGF, Vascular Endothelial Growth Factor; n.a, not available; CI, Confidence interval. Particulate matter from urban samples was understood as a mixture of exhaust and non-exhaust emissions.

### 3.3 Fetal and maternal vascular alteration by particulate matter exposure

Translocation of inhaled PM2.5 into the systemic circulation has been associated with vascular endothelial cell damage, promoting increased risk of cardiovascular disease (105, 106). The most prominent mechanisms associated with PM_2.5_ health effects are oxidative stress and inflammation (**Table 1**) (107).

This crosstalk between altered redox homeostasis and inflammation-related pathways has been termed “oxi-inflammation” to describe the pre-pathological condition (108). This issue requires a special attention for pregnant women since normal pregnancy itself is characterized by systemic inflammatory activity and the placenta is a great source of reactive oxygen species (ROS) (109). In this regard, increased levels of 8-hydroxy-2′-deoxyguanosine (8-OHdG), an indicator of ROS-mediated mitochondrial damage, have been found in both maternal and cord blood during pregnancy in women exposed to both PM_2.5_ and PM_10_ (92). This has been interpreted as that exposure to air pollution in the first years of life plays an important role in the appearance of oxidative stress, both at the mitochondrial and systemic level (92). Furthermore, high levels of 8-OHdG in the cord blood increase the probability of intrauterine growth restriction as compared with newborns below the median level of mitochondrial damage (110). Moreover, HUVEC incubation with 10 μg/cm^2^ of PM_2.5_ induces alterations in the mitochondria, leading to an increase in the mitochondrial fusion gene Mfn1 and a decrease in the fission genes Opa1 and Drp1 (87). Authors suggest that fusion-fission imbalance is associated with mitochondrial dysfunction that could induce cardiovascular disease (87), and during pregnancy this imbalance has been associated with preeclampsia (111, 112). *In vitro* experiments in HUVEC demonstrated that PM_2.5_ increases the expression of adhesion proteins (ICAM-1 and VCAM-1) and decreases the expression of a tight junction protein, zonula occludens-1 (ZO-1) leading to endothelial activation and increases the endothelial barrier permeability, respectively (85, 113). In this experimental setup, PM_2.5_ triggers the secretion of inflammatory interleukins such as IL-6 and IL-1β, increasing the inflammatory response (85, 86). A breakdown of intercellular junctions and increased adhesion molecules are characteristic events of inflammation and endothelial dysfunction, which are observed in pregnancy pathologies such as gestational diabetes, preeclampsia and obesity (114). On the other hand, the endothelial damage related to PM_2.5_ exposure involves endothelial-mesenchymal transition (EndMT), triggered by the activation of the transforming growth factor-β (TGF-β) pathway, linked to high levels of reactive oxygen species (ROS) and PAHs (88, 115). Interestingly, it has been reported that EndMT contributes to the development of atherosclerotic lesions, which could explain the role of air pollution in the development and progression of cardiovascular disease (116), where upregulation of angiotensinogen and the angiotensin-converting enzyme has been shown, resulting in increased circulating angiotensin II and activation of the angiotensin II receptor type 1, thus favoring vascular contractility (89, 117). Furthermore, exposure to PM_2.5_ causes a reduction in the bioavailability of NO in the vessel wall, impairing the endothelial-dependent vasorelaxation (90). On the other hand, a study performed in children (7.9 ± 1.3 years of age) chronically exposed to outdoor air pollution showed increased levels of circulating endothelin-1 (ET-1), a potent vasoconstrictor, with a positive correlation with PM_2.5_ exposure levels (91). However, a study conducted in young healthy adults who were exposed to natural variations in PM_2.5_ showed a negative correlation between ET-1 levels and PM_2.5_ exposure levels (118). Another mechanism that could explain the endothelial dysfunction induced by PM2.5 exposure, is the interleukin 22/interleukin 22 receptor (IL22/IL-22R) pathway (119, 120). The exposure to PM_2.5_ activates the AhR in circulating innate lymphoid cells and induces cytokine IL-22 gene expression (121). IL-22 is a cytokine that plays pro- and anti-inflammatory functions, through interactions with hematopoietic cells, such as macrophages and with endothelial and epithelial cells (122). Endothelial cells express IL-22R, the interaction of IL-22 with its receptor can induce the production of adhesion molecules, endothelial activation, and the secretion of numerous proinflammatory mediators (123). Increased IL-22 has been observed in the blood of pregnant women with preeclampsia and premature rupture of membranes (124, 125).

### 3.4 Placental tissue and particulate matter exposure

It was reported that black carbon particles from air pollution can translocate from the maternal lungs into the maternal circulation reaching the placenta (17). The PM_2.5_ exposure during the first and second trimesters of gestation has been positively associated with the amount of 3-nitrotyrosine (3-NTp) in the placental tissue from 330 mother-newborn pair cohorts (93). 3-NTp is a well-known biomarker of peroxynitrite because of its positive association with the rate of protein degradation and therefore for a biomarker of both nitrosative and oxidative stress and inflammation (126).

On the other hand, placental methylation status of circadian pathway genes (CLOCK, BMAL1, NPAS2, CRY1-2, and PER1-3) were positively and significantly associated with intrauterine PM2.5 exposure during the third trimester (127). Genetic abnormalities in the molecular circadian pathway have been associated with chronic noncommunicable diseases, such as obesity (128), metabolic syndrome (129) and diabetes (130). It has been reported that the accumulation of PAHs in the placenta from healthy pregnancies decreases the presence of PAHs in fetal blood, suggesting that under normal conditions the placenta acts as a reservoir decreasing the transfer of PAHs from the mother to the fetus (40). While, in placental tissues from pregnancies associated with hypertensive disorders, diabetes, or preterm delivery, PAHs concentration decreases together with an increase of PAHs in fetal blood (40). Familari and colleagues (94) reported that the exposure of HTR-8/SVneo cells (immortalized first trimester trophoblast cell line) to urban pollution particles led to reduced cellular growth, increased proinflammatory cytokines (IL-6), upregulated expression of endocytosis and intracellular transport proteins, as well as an alteration in the amino acid metabolism and autoimmune responses. Similarly, another study showed that the cell line exposed to PM_2.5_ for 48 h, undergoes cytotoxicity, diminution of hCG secretion, and an increase of IL-6 production (95). The exposure of these cells to PM_2.5_ (120 µg/ml) induced cell-cycle arrest and inhibited migration and invasion of HTR-8 cells by up-regulating the expression of tissue inhibitors of metalloproteinases (TIMP1 and TIMP2) and down-regulating Collagen I expression (96).

Elevated levels of B[a]P in placental tissue from women with preterm delivery shows a significant correlation with lower glutathione (GSH) levels and higher levels of thiobarbituric acid-reactive substances (TBARS) in this tissue (39). This has been taken as an indication of a possible contribution of PAHs in preterm delivery through redox imbalance (39). Moreover, PAHs exposure (between 0.24 and 2.47 ng/m^3^), during the third trimester of pregnancy, was associated with lower global DNA methylation in umbilical cord white blood cells (97). Additionally, the presence of detectable PAH-DNA adducts in cord blood was shown to be positively associated with global methylation levels. Since both global hypomethylation and hypermethylation of specific genes have been associated with cancer and other diseases in humans, it is remarkable that maternal PAHs exposure can modify genomic DNA methylation status in the fetus (97). In this regard, the presence of PAHs from air pollution in placental tissue is inversely associated with placental weight and cord length (38).

The PAHs are linked with endocrine disruption in trophoblast cells, since a steroidogenic enzyme, aromatase that catalyzes the aromatization of fetal and maternal androgens into estrogens is inhibited in placental JEG-3 cells exposed to organic extracts from biomass burning collected in winter (98). The most abundant and studied PAHs, B[a]P, enters human cells and is metabolized by cytochrome P450 1A1 (CYP1A1) into different compounds. The benzo(a)pyrene-7,8-dihydrodiol-9,10-epoxide (BPDE) metabolite can cross the placenta and reach the fetal compartments leading to toxicity and DNA damage through the generation of BPDE-DNA adducts (43). In addition, the incubation of human trophoblast cells Swan 71 with 2.0 µM of BPDE for 24 h reduces the human chorionic gonadotropin (hCG) secretion, promotes the increase of pro-inflammatory cytokines IL-6 (27.5-fold), TNF-α (51.9-fold), and induces mitochondrial fragmentation and dysfunction due to an increase in mRNA and protein levels of mitochondrial fission genes in these cells (99). On the other hand, JEG-3 cells exposed to PM_2.5_ increase their hCG secretion, and an important inhibition of progesterone synthesis, which could be an indication that PM_2.5_ may directly inhibit the phosphorylation status of Protein Kinase A in JEG-3, with a concomitant inhibition of the protein expression in progesterone-synthesis, leading to a suppression of the progesterone levels (100). Furthermore, placental JEG-3 cells exposed to 10 μM B[a]P for 72 h leads to cell cycle arrest (G2/M phase) and a significant decrease in cell proliferation, likely through the phosphorylation of histone H2A variant H2AX (γ-H2AX) (101).

The exact concentration of PM_2.5_ that reaches the placenta of a woman exposed to air pollution is unknown. It is not valid to convert from the unit used in most experimental studies (μg/ml or ng/ml) to the unit reported by air quality sensors (μg/m^3^). However, one study indicates that levels of 5000 ng/ml (5 μg/ml) could correspond to 25 μg/m^3^ (95). The same study indicates that 10,000 ng/ml (10 ug/ml) could correspond to levels that can be observed in a more polluted city with PM_2.5_ concentrations of 50 μg/m^3^ (95). The reference value for 24 h exposure to PM_2.5_ established by WHO global air quality guidelines is 15 ug/m^3^ (7, 131).

### 3.5 Pregnancy outcomes and indoor air pollution

The main literature on-air quality-related mortality is focused on pollutant measurements taken outdoors. However, the influx of outdoor air influences indoor air quality, which already includes specific indoor emissions sources, relationships between building systems/construction methods, and occupant behavior (7). According to the United States federal government agency called Environmental Protection Agency (EPA), the levels of indoor air pollutants are often 2 to 5-fold higher than outdoor levels (23). Since people spend most of their time inside, indoor conditions play a prominent part in the overall human exposure to air pollution (132). This is particularly relevant since pregnant women spend most of their time indoors, especially toward the end of pregnancy (133). Indoor pollutants are mostly caused by human interaction at home and in classrooms, but they can also be found in daycare centers, social entertainment settings, and micro-environments, including automobiles, buses, trains, and airplanes (132, 134). Indoor air pollutants can become outdoor air pollutants, resulting in the so-called “neighborhood” pollution effect (132); this is especially relevant in disadvantaged neighborhoods that may use biomass for cooking or heating, which increases the concentration of pollutants in their living area, compared to people living in more socioeconomically advantaged neighborhoods (135). Allergens, mainly house dust mites and insects, pollen, animal sources, molds, and bacterial endotoxins, are examples of biological indoor air pollutants (136). Chemical air pollutants such as gases, particulate matter, formaldehyde, and volatile organic compounds (VOCs) are also present (134). The latter derive from various sources, the most common indoor are burning wood, household chemicals (disinfectants, bleach, dry cleaning fluid, aerosols, air fresheners, paint, varnish, and pesticides), also incense, candles, and cooking (137–140). Cooking with polluting fuels such as gasoline, kerosene, and biomass (wood, charcoal, crop residues, and animal manure) causes household air pollution, which is a global environmental health problem (132). There is evidence that the exposure to labeled carbon particles (less than 100 nm) for 1 min is sufficient to appear in the blood of healthy volunteers and to remain detectable for 60 min (141). However, there are very few studies that have evaluated the effect of indoor exposures to PM_2.5_ on pregnancy and delivery outcomes (142–144). A predominantly indoor air pollutant, formaldehyde has been associated with reduced birth weight (142). The kinetics of transplacental transfer (from the maternal to the fetal compartment) of formaldehyde was studied with a perfused human placental cotyledon model, showing that the compound can accumulate in the placenta and fetus (102). In addition, formaldehyde exposure reduced the synthesis and secretion of the peptide placental hormones (pGH: placental growth hormone, hPL: human placental lactogen, and hCG: human chorionic gonadotrophin), a fact that appears to be mediated by oxidative stress, since hCG production was restored by n-acetylcysteine (102). The cooking oil fumes-derived PM_2.5_, is another source of indoor air pollution that has been associated with preterm birth (145) and low birth weight (57). Human umbilical vein endothelial cells (HUVEC) exposed *in vitro* to PM2.5 derived from cooking oil fumes lead to overproduction of ROS, inflammation, and inhibition of angiogenesis (103).

A study conducted on 68 pregnant women using kerosene stoves, found that cooking with kerosene is associated with reduced birth weight and low levels of micronutrients such as iodine, vitamin B6 and homocysteine in mothers and newborns (146). Solid fuel for cooking has been associated with an increased risk of cesarean delivery, low birth weight, neonatal mortality, and acute respiratory infection among children (147). While another study performed on 695 pregnant women using biomass fuels for cooking in Temuco (Chile) and Bariloche (Argentina), found no association between perinatal morbidity (pre-term birth and low birth weight) and household air pollution exposure; the study highlights that these results may be related to the fact that the studied population cooks in ventilated rooms compared to other studies (148). The link between poor ventilation and persistent indoor air pollution has been explored by different studies, suggesting its association with the development of adverse pregnancy outcomes (147, 149–151). The assessment of exposure to indoor air pollution during intrauterine life has some limitations. For example, some studies have evaluated the personal exposure of pregnant women to various pollutants, however, they do not distinguish indoor and outdoor concentrations of these pollutants and therefore cannot specifically explore associations between indoor air pollutants and birth (142, 152). In this regard, an attempt has been made to evaluate compartmentalized exposure, using passive sampling techniques and surveys that include questions about the types of chemicals used to clean, the type of kitchen, and the time spent indoors (142). When analyzing the personal variability of indoor and outdoor exposures, some of them may be misclassified, furthermore, in many cases, seasonal changes and spatio-temporal variability are not considered during pregnancy exposition measurements (153, 154). The source of individual pollutants is challenging; therefore, it is necessary to apply integrated approaches (*i.e.* survey and exposure models) (155).

## 4 Mitigation strategies

**Figure 1:**
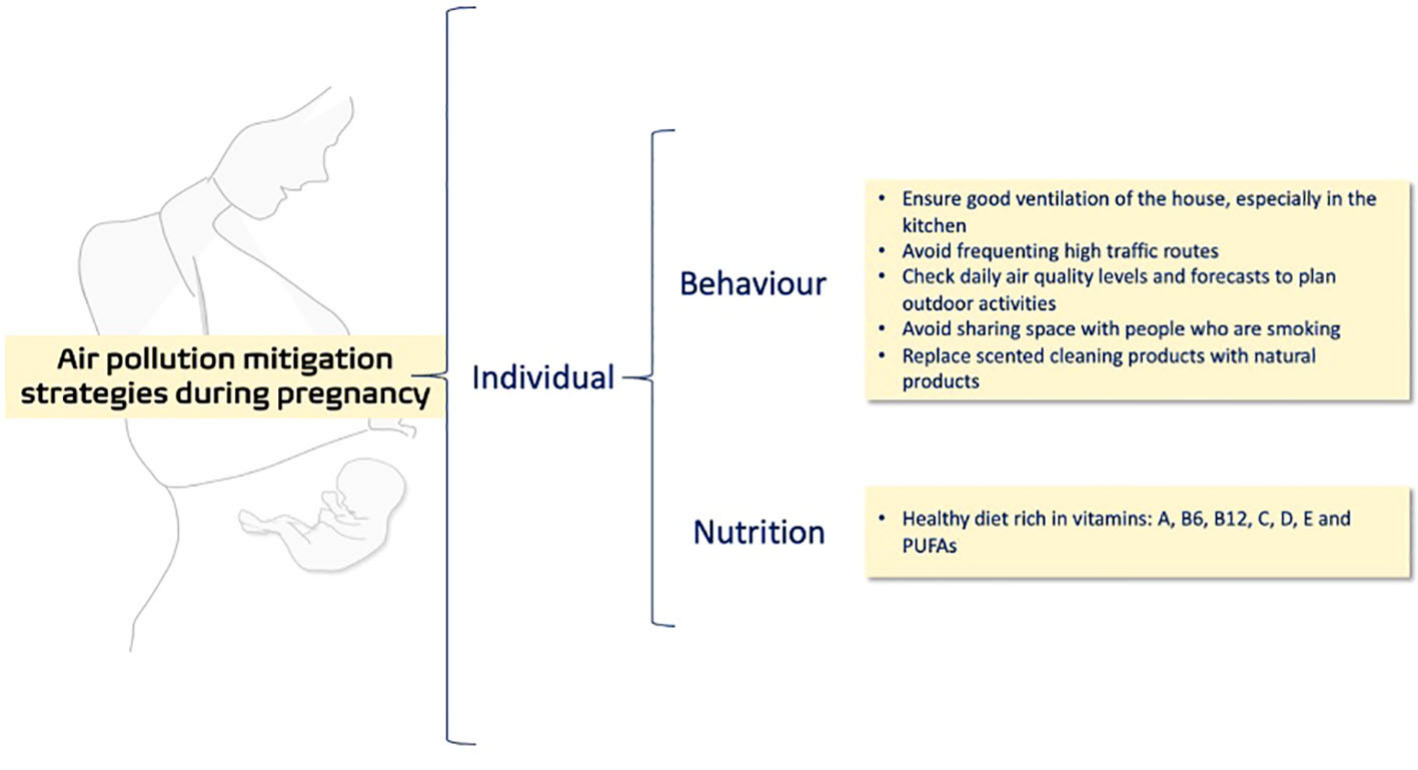
Proposed approaches to reduce exposure to air pollution during pregnancy.

The most obvious way to avoid the deleterious effect of air pollution is not to be exposed to it. However, this recommendation is not practical since everyone must breathe the available air. We need to reduce the air pollution in our cities, but unfortunately is a long-term process that demands a government commitment, along with education and other strategies related to reduce the sources of pollution. While in some cities it is possible to avoid busy roads or highways at least during pregnancy, however in many cities this is not possible. Thus, individual and public strategies focused to reduce the harmful effects of the available polluted air are needed (**Figure 1**).

### 4.1 Behavior actions

Reducing exposure to air pollution during pregnancy is a key opportunity to provide better health to the child (156). A primary prevention measure that can be given to the mother at prenatal care, is to ensure good ventilation to reduce the exposure to indoor air pollution (157). One of the biggest sources of pollution in homes is the use of the kitchen. To reduce or to eliminate air pollution in the kitchen, the American Lung Association recommends using the exhaust fan and ensuring ventilation in the kitchen (158). Another recommendation made by the same association is to get rid of fragrant and scented products such as air fresheners and cleaners, replacing them with natural products such as vinegar, peroxide, and baking soda, or non-toxic brands. Another individual measure is to check daily air quality levels and to perform outdoor activities when pollution levels are lower. It is also recommended that pregnant women avoid sharing space with people who are smoking.

### 4.2 Nutrition

Since the imbalance in oxidant production is one of the prominent mechanisms leading to cellular damage linked to air pollution, the presence of antioxidants from nutrition represents an opportunity to mitigate the air pollution effects (159–164). According to the Food and Drug Administration antioxidants are substances that, following absorption from the gastrointestinal tract, participate in physiological, biochemical, or cellular processes that inactivate or prevent free radical-initiated chemical reactions (165). The common antioxidants from diet or supplementation, are DL-alpha-tocopherol acetate (vitamin E), ascorbic acid (vitamin C), beta-carotene (vitamin A), omega-3 polyunsaturated fatty acids (omega-3), and selenium, that has been reported to be involved in the deactivation of free radicals (166, 167). About the antioxidant air pollution mitigation, a study conducted in individuals chronically exposed to PM_2.5_ shows that supplementation with omega-3 could modulate the plasma levels of cellular redox systems by increasing glutathione (GSH) and Cu/Zn superoxide dismutase (SOD) activity (168). Additionally, another study shows that oxidized low-density lipoprotein (OxLDL) decreased following the fish oil supplementation in a cohort of healthy university students exposed to the average level of PM_2.5_ of 38 ug/m^3^ for four months (169). Simultaneous treatment of human umbilical vein endothelial cells (HUVECs) with vitamin E and PM_2.5_ protects against the reactive oxygen species (ROS) production and the increased levels of lipid peroxidation (161). An epidemiologic cohort study found that women in the first and second trimesters of pregnancy with lower vitamin A intakes have higher negative effects on birth weight due to prenatal PM_2.5_ exposure than women with higher intakes (170). Indeed, concurrent air pollution and poor nutritional status are associated with adverse health and pregnancy outcomes such as low birth weight and preterm birth (171). The lack of vitamin D is mainly due to low exposure to ultraviolet B (UVB) radiation since the skin synthesis provides 90% of all the body’s requirements (172). The levels of air pollution influence the percentage of the ground level of UVB (173). In this way, a longitudinal cohort study conducted in 3285 pregnant women, found that the PM_2.5_ exposure during the third trimester and the entire pregnancy was inversely associated with 25(OH)D levels (174). In addition, the mediating effect of total net daily UV-B radiation (radiation reaching ground level) on the inverse association between prenatal PM2.5 exposure and maternal circulating 25(OH)D levels was 70% (174). Similar results were found in a study performed in 375 mother-child cohorts. It was found that the exposure to ambient urban air pollution during late pregnancy may contribute to hypovitaminosis D in the offspring and suggest that this factor could affect the child’s risk of developing diseases later in life (175). Epidemiological data associates the presence of urban particulate matter in polluted air with respiratory diseases, and vitamin D deficiency (176). This could be linked to the induction of a proinflammatory and potentially pathogenic T helper 17 cell (Th17) profile (176). Lower levels of vitamin D could increase the risk of low birth weight (177, 178). In addition, the incidence of asthma linked to air pollution exposure is higher among low-term-birthweight children (179). It has been proposed that restoring levels of vitamin D may mitigate the urban particulate matter adverse effect associated with respiratory health (176).

On the other hand, a study found that maternal exposure to NO_2_ from traffic-related air pollution, along with low dietary intake of methyl nutrients such as folate, vitamins B6 and B12 are related to the greatest odds of congenital heart defects (180). Recent experimental evidence suggests that vitamin B (folates, vitamin B12, and B6) supplementation in healthy non-smoking volunteers could mitigate the effect of PM_2.5_ exposure on cardiac autonomic dysfunction and inflammation (181). More data of the same studied population showed that vitamin B supplementation prevents alterations in mitochondrial DNA content in circulating CD4^+^ Th cell induced by PM_2.5_ exposure (182). Folate (vitamin B9) and vitamin B12 (cyanocobalamin) are hydrosoluble vitamins naturally present in some foods, added to others, and in the dietary supplement (183). Folate together with vitamin B12, acts as a coenzyme in the metabolism of 1-carbon compounds, required for numerous cellular functions such as *de novo* synthesis of purines, thymidylate, and the generation of the methyl groups for the methylation reactions of DNA, RNA, proteins, and lipids (184, 185). Considering all this information, monitoring the levels of vitamin D as well as those of B12 during pregnancy should receive more attention in clinical practice. An important educational campaign could be aimed at raising awareness among pregnant women about the importance of dietary practices to mitigate the health risks of air pollution (164).

## 5 Concluding remarks

This study reviews the experimental evidence on the effects of indoor and outdoor pollution during pregnancy and discusses some mitigation strategies. The deposition of air pollutants on the air-blood barrier triggers oxidative stress and inflammation, during pregnancy increasing the risk of developing complications that affect the health of the mother and the offspring. Mitigation strategies should include advice to pregnant women on ways to reduce exposure to indoor and outdoor pollution and the importance of this issue during pregnancy. There is a knowledge gap regarding the effects of non-exhaust emissions on intrauterine development. Greater efforts and interaction between different disciplines are needed to develop effective prevention and risk assessment strategies that can significantly reduce the adverse effects of air pollution during pregnancy.

## Author contributions

DC, JG, and JU contributed to conception and design of the study. DC and AG organized the database. DC and JU wrote the first draft of the manuscript. RM, IC-W, MF, FG, SI wrote sections of the manuscript. All authors contributed to the article and approved the submitted version.
